# Neurobrucellosis with ischemic stroke and spinal cord involvement: a case report

**DOI:** 10.1186/s12883-021-02155-2

**Published:** 2021-03-20

**Authors:** Hongfeng Wen, Di Jin, Lina Cai, Tao Wu, Haichao Liu

**Affiliations:** Department of Neurology, Aerospace Center Hospital, 100049 Beijing, China

**Keywords:** Neurobrucellosis, Stroke, Spinal cord, Cerebrospinal fluid

## Abstract

**Background:**

Brucellosis is a common zoonotic disease that may have a variety of clinical manifestations when it affects the nervous system. Ischemic stroke is a rare clinical symptom, but if it is not diagnosed and treated early, it may cause more severe consequences.

**Case presentation:**

We report a 38-year-old man presenting with hearing impairment for four years and sudden weakness of the right limb for two years, recurrent aphasia, and gradual weakness of bilateral lower limbs for nine months. He had bilateral positive Babinski’s sign. Cerebrospinal fluid (CSF) showed raised protein and pleocytosis. Magnetic resonance imaging (MRI) showed ischemic infarcts in the pons and extensive enhancement of spinal meninges combined with spinal cord atrophy and ischemia. The tests revealed Brucella Rose Bengal positive in serum and CSF. Brucella culture in CSF was also positive. Next-generation sequencing (NGS) of CSF revealed positive for Brucella with 105 species were detected. He showed significant improvement with antibiotics at five months follow-up.

**Conclusions:**

Neurobrucellosis may mimic stroke and transverse myelitis like syndromes. NB is a treatable infectious condition and should always be considered in the differentials, especially if there are risk factors, as in our case.

## Background

Brucellosis is a common zoonotic disease, usually caused by infections of different Brucella species. Domestic animals such as cows, sheep, goats, camels, horses, swine, cats, and dogs are the most common sources of infection. Human brucellosis is not usually acquired through animal contact but is transmitted more often by consumption of infected livestock products [1], such as infected milk or meat. Brucellosis infection of the central nervous system (CNS) is a rare but severe complication [2, 3] and the varied clinical manifestations, including confusion, meningoencephalitis, myelitis, peripheral or cranial neuropathies, and psychiatric manifestations, make the diagnosis challenging. When Brucella irrupts cerebrolvascular, it can be manifested as ischemic infarction and transient ischemic attack, which are also relatively rare symptoms. We report a case of neurobrucellosis (NB) mimicking stroke and transverse myelitis. This case was diagnosed by NGS of the CSF and subsequently confirmed by Brucella cultured from CSF.

## Case presentation

A 38-year-old man had a history of back and migratory joint pain for seven years, gradual bilateral hearing loss for four years, tinnitus for three years. In 2017, he admitted to another hospital because of sudden weakness in his right limbs and diplopia. Cranial magnetic resonance imaging (MRI) revealed one lesion located in pons, which was treated as ischemic stroke. One year later, he reported two similar episodes of recurrent language comprehension and expression difficulties. Each episode lasted for approximately 6–12 h, and MRI showed no new lesions, which was interpreted as a transient ischemic attack (TIA). Fifteen months after the first stroke attack, the patient noticed progressive bilateral lower limb weakness until unable to walk. Other clinical manifestations included memory disturbances for one year, recurrent episodes of high-grade fever for eight months, dysuria, and difficulty in defecation for two weeks. He had lost about two to three kilogram weight since the stroke onset. The patient suffered a car accident in 2010 and there were no clear complications. He had a past medical history of hypertension that was well-controlled with medication. His daily medical therapy included aspirin and clopidogrel as prophylaxis for the neurological symptoms that had been ascribed to a cerebrovascular event.

**Fig. 1:**
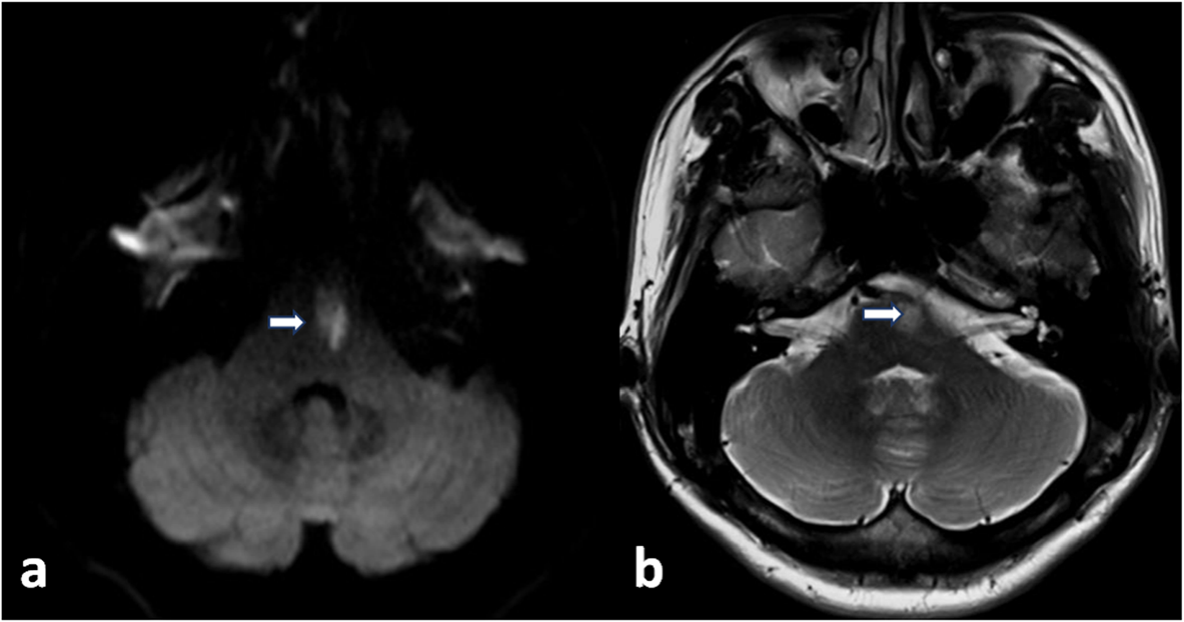
Diffusion weighted imaging (**a**) and T2-weighted image (**b**) showed pons infarction (arrow)

**Fig. 2:**
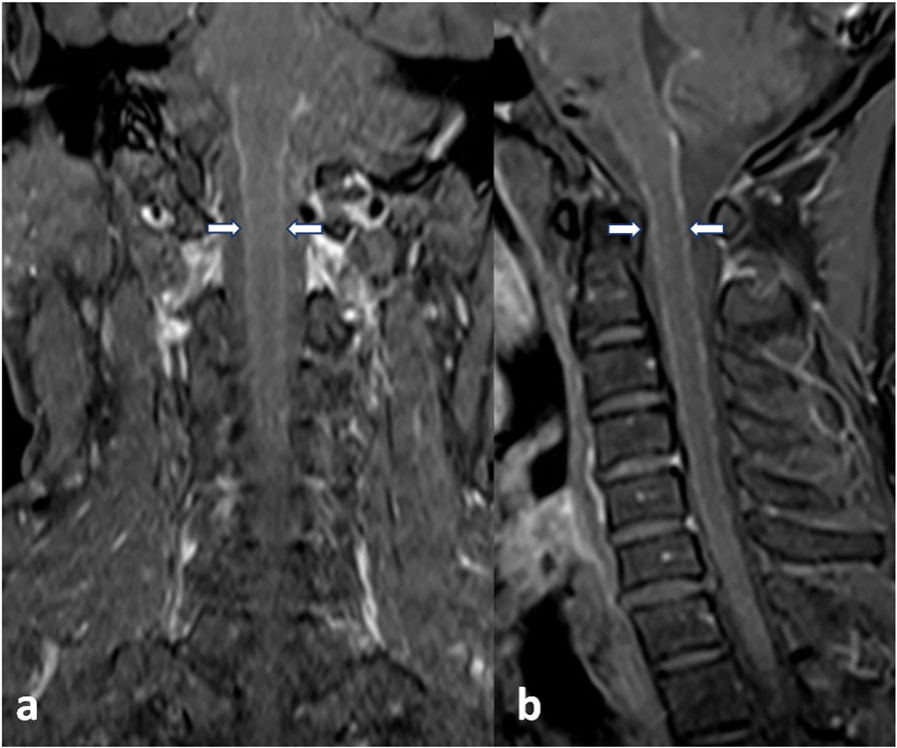
In postcontrast coronal (**a**) and sagittal series (**b**), diffuse leptomeningeal enhancement (arrow) of the cervical spinal cord were observed

**Fig. 3:**
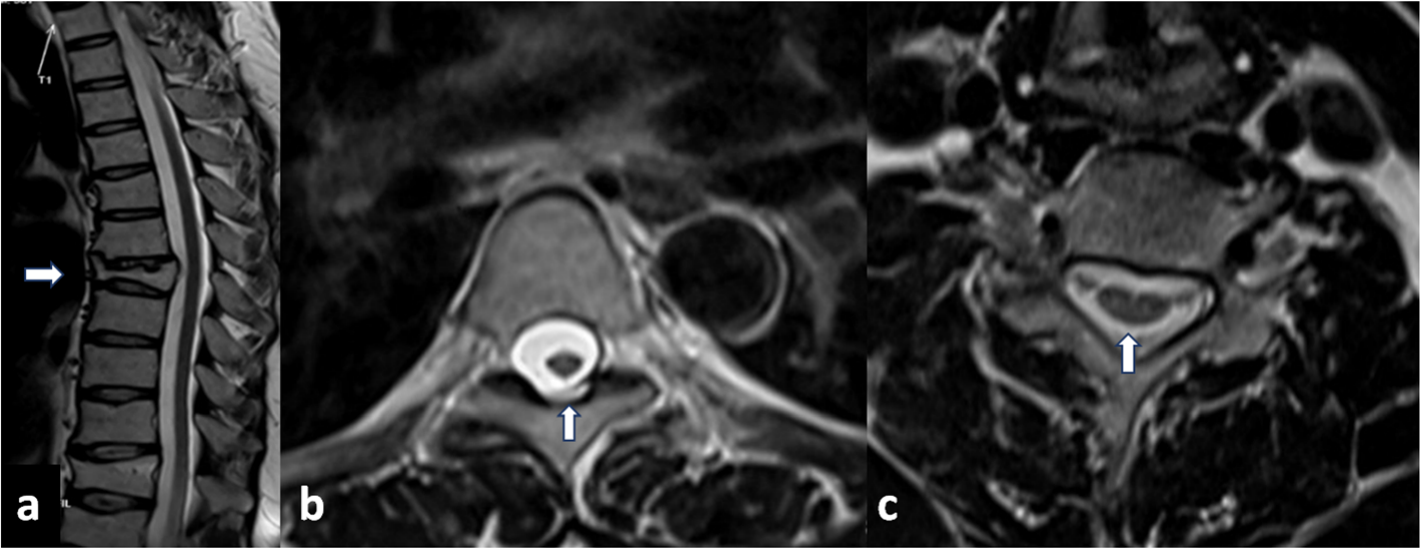
T2-weighted images view compression fracture of T7 vertebrae (arrow) in dorsal spine-sagittal (**a**), atrophy of thoracic spinal cord (**b**), and “Snake eyes sign” (means ischemia) of the cervical spinal cord (**c**) in the axis

The neurological examination at this admission disclosed left central facial paralysis. There was no abnormalities of extraocular movements. His left upper extremity muscle strength was normal, with right upper extremity was 4/5 grade, and both lower limbs were 2/5 grade. Deep tendon reflexes were brisk in the right upper limb and bilateral lower limbs. The patient denied dissociated sensory loss or sensory level. Bilateral Babinski’s signs were positive. There were no meningeal signs. Moter and sensory peripheral nerve conduction had no obvious foundings. Tone audiogram detected the presence of a slight sensorineural hearing loss in biliteral ears (40-60dB). During his admission, he developed low fever that fluctuated between 37.3℃ to 39℃. The routine blood tests, anti-Streptolysin O and erythrocyte sedimentation rate were normal. Some chronic infections tests such as syphilis, tuberculosis, and fungal infections were negative. Ultrasonography detected enlargement of neck and inguinal lymph nodes. The previous cranial MRI (in year 2017) had shown lesion located in pons interpreted as a vascular disease (Fig. 1). Contrast-enhanced MRI (in year 2019) of the cervical spinal cord demonstrated diffuse leptomeningeal enhancement (Fig. 2). T2-weighted images showed T7 vertebral compression fracture, with thoracic spinal cord atrophy and cervical spinal cord ischemic changes. (Fig. 3). A lumbar puncture on admission revealed mildly elevated pressures (20 cm H_2_0). He denied a lymph node biopsy for invasive reason.

**Fig. 4:**
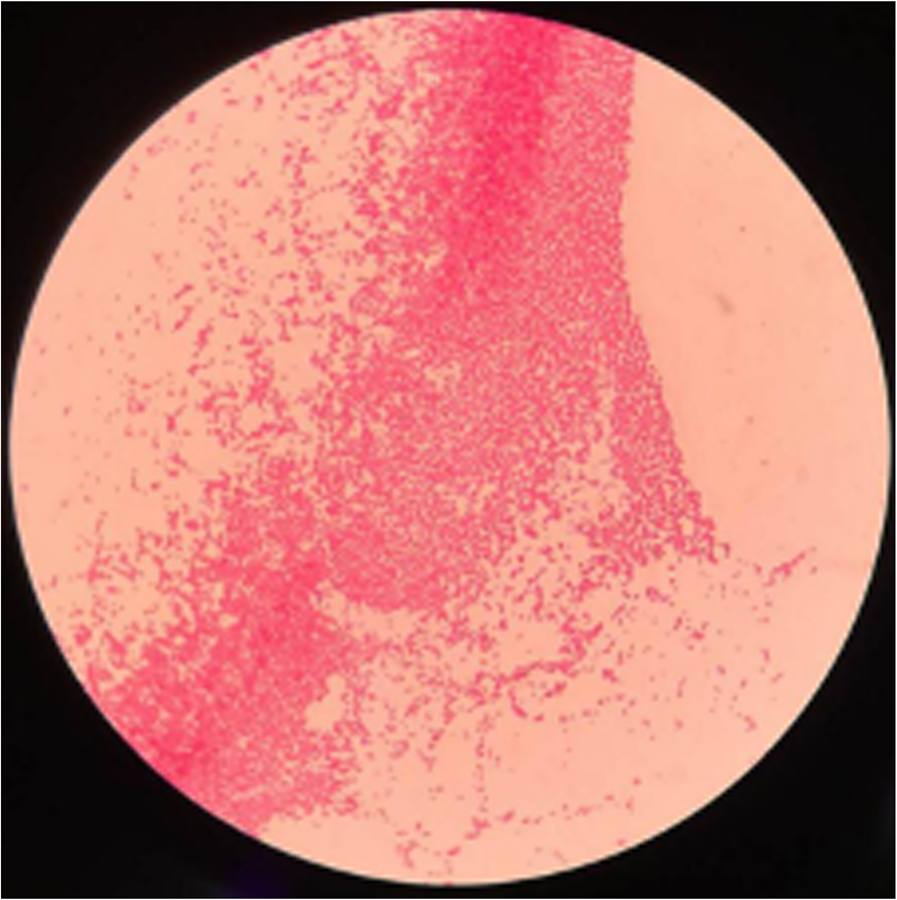
Gram-stained Brucella colonies of cerebrospinal fluid culture under an optical microscope

The CSF analysis showed elevated leukocyte, which consists of 120/ul mononuclear cells with 72 % lymphocytes prominently (0–10/ul). The protein was 219.30 mg/dL (15.00–45.00 mg/dL), chloride was 118.9 mmol/L (120.0-130.0 mmol/L) and glucose was 2.40 mmol/L (2.40–4.50 mmol/L). Given the patient’s history of raising swine and drinking unpasteurized goat milk for several years before the onset of the stroke, we did specific laboratory tests for him. The Brucella Rose Bengal test of blood and cerebrospinal fluid were positive. Next-generation sequencing (NGS) of the CSF found the sequence corresponded to Brucella was 105 species. Furthermore, CSF culture was positive in the following ten days (Fig. 4).

**Fig. 5:**
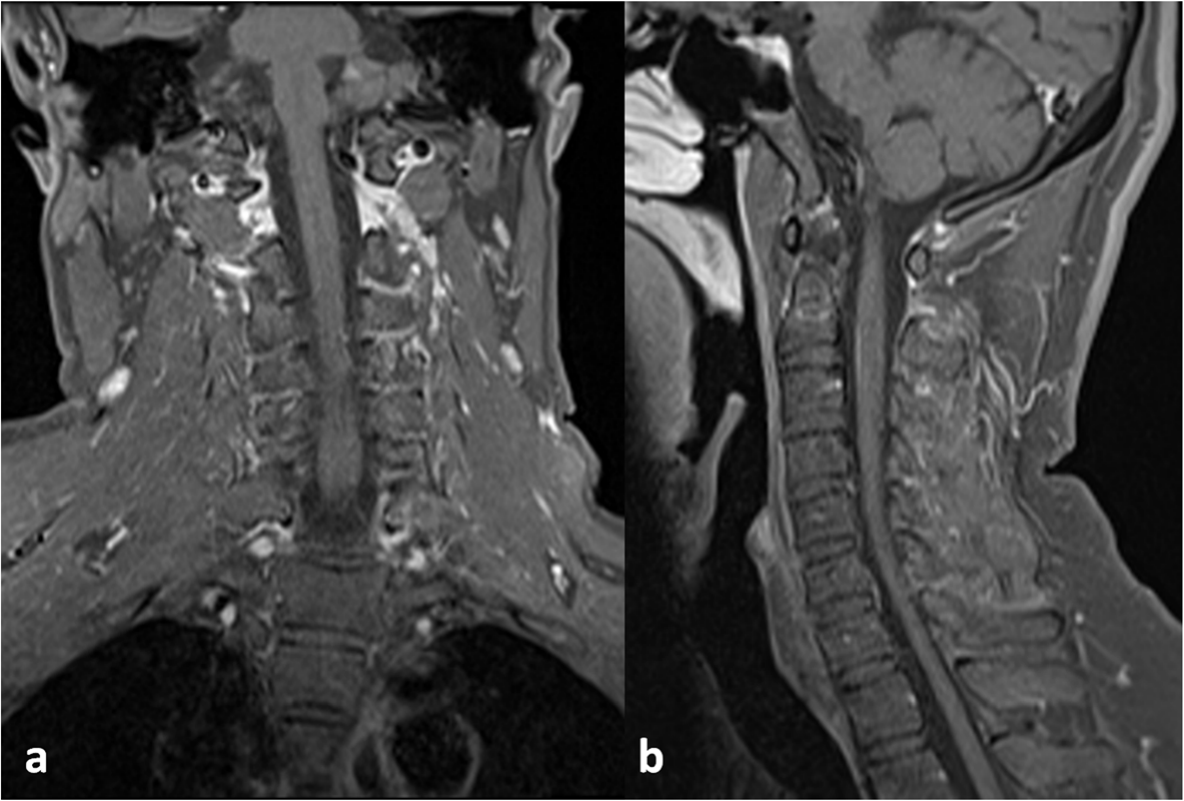
Enhancement of cervical MR at ten-month follow-up showed that the leptomeningeal enhancement was significantly improved compared with the previous one. There was no obvious enhancement in the coronal view (**a**), and slightly enhancement was seen in the sagittal view (**b**)

The treatment of intravenous administration of Ceftriaxone (2 g twice-daily), oral administration of minocycline (100 mg twice daily), and rifampicin (600 mg once daily) were started. About one week, the hearing loss and dysuria were significantly improved. At one-month follow-up visit, the patient stopped ceftriaxone and continued to use oral antibiotics erratically. At ten-month follow-up, he was able to walk independently with the MRC grade of 3/5 in the affected extremites. CSF analysis revealed the cell count, glucose and chloride level in normal range, and a little elevated with protein (80.20 mg/dL). Tone audiogram showed that his hearing in both ears had recovered to 20–40 dB. Contrast cervical MR imaging found slightly leptomeningeal enhancement which was better than the first scan (Fig. 5).

## Discussion and conclusion

Brucellosis is the most common zoonotic infection in the world. The involvement of the nervous system is known as ‘neurobrucellosis’(NB). The frequency of NB with CNS involvement is approximately 5 %-7 % of all cases of brucellosis [3, 4]. The diagnosis of NB is difficult because its clinical manifestations are non-specific, and the sensitivity of routine culture tests is low. Manifestations of NB include meningoencephalitis, cerebrovascular disease, peripheral and cranial neuropathies, or myelitis [5]. In the study of Gül et al., the most common symptoms of NB were headache (57 %), fever (57 %), sweating (30 %), weight loss (28 %), and back pain (23 %); weakness (15 %)and hearing loss (10 %) were found to be rare symptoms [5]. Our patient presented with hearing loss, back and joint pain, weakness, fever, and dysuria, which is consistent with previous research results. Although cranial nerve involvement is unexpected in NB, the most affected cranial nerve is the vestibular cochlear nerve due to vascular spasm or endotoxin [6, 7]. Our case had hearing impairment as the initial manifestation and progressed to a moderate loss. The MRI revealed the enhancement of bilateral auditory nerves, which also supports the presence of the impairment of VIII cranial nerves. The mortality rate of NB in the post-antibiotic era is 0-5.5 %, but permanent neurologic deficits, especially deafness, are common (20–30 %) [8]. Because the first time the case was admitted to the hospital for stroke, other medical histories collection were not detailed, which led to the delay of diagnosis. However, after a course of antibiotics treatment, his hearing loss was also significantly improved.

The mechanisms of stroke in the NB was considered as vasculitis of small vessels. Vascular events secondary to inflammation may cause lacunar infarcts, hemorrhagic strokes, or venous thromboses [9]. Different mechanisms related to the cerebrovascular involvement of brucellosis have been described [10]. Some cases of cerebral vasculitis findings in MR angiography or digital subtraction angiography have been reported. However, the cerebrovascular can also be completely normal, according to the previous reports [11, 12]. MR angiography and CT angiography of our case were normal.

Neuroimaging studies of neurobrucellosis varies significantly among individuals [9]. The most common imaging abnormalities are meningeal enhancement, white matter change and vasculitis [10]. Myelitis or myeloradiculopathy is very rare to happen and reported in only 1.2 to 3 of% cases of neurobrucellosis [13]. Brucella may cause various spinal impairment including spondylitis, spondylodiscitis and/or discitis. The mechanisms are varied, which include the infectious process itself, immune mechanisms, septic embolization, or venous thrombosis due to brucella vasculitis. Spondylitis is inflammation and infection of vertebrae which most common affected in lumbar spine and sacrum. However, due to his previous history of car accidents, it is difficult to determine whether the vertebral fracture was caused by trauma or vertebral osteomyelitis caused by brucellosis. Our case presented with myelitis symptoms and suspicious spondylitis (Fig. 3a). Considering that the middle thoracic vertebral is not a common site of spondylitis caused by Brucella, and there is no significant spine compressed or degenerated at the corresponding level, we speculate that the lesion in T7 vertebral is more likely to be caused by trauma. Besides, the atrophy of the thoracic spinal cord (Fig. 3b), the ischemia of the cervical spinal cord (Fig. 3c, the snake eyes sign), and diffuse enhancement of the spinal meninges may involve bilateral pyramidal tract dysfunction, which becomes possible explanations for the progressive weakness of the lower extremities and dysuria.

The clinical presentation of NB is quite varied and can mimic many other diseases. For this reason, laboratory test results of blood and CSF are essential to the differential diagnosis of chronic infections such as tuberculosis and Lyme disease, especially in endemic areas. The detection of specific antibodies usually confirms the diagnosis of NB in blood or CSF by enzyme-linked immunosorbent assay (ELISA) or Coombs’ test, or less commonly by positive CSF cultures [14]. Although tissue culture is the gold standard for diagnosis of brucellosis, due to its long culture period and low sensitive rate, it may delay the diagnosis of patients [15, 16]. Our case underwent two CSF cultures, and the second one was positive, it took seven days. Serology is more sensitive for detection, but it can lead to false-positive results and may not distinguish between active and prior infection. Molecular methods based on the detection of nucleic acid such as PCR [17] and now potentially metagenomic NGS [18] can offer increased sensitivity and specificity over conventional diagnostic testing. As a new technique, NGS is increasingly used for the clinical diagnosis of infectious diseases of the central nervous system [19–22]. The rapid diagnosis led to prompt treatment with the appropriate antibiotics. We present the use of the NGS assay to diagnose NB, and the result was confirmed by the Brucella Rose Bengal test and CSF culture.

Although there is no specific guidance in the treatment of NB, many authorities maintain the combination regimen of doxycycline and two or more antibiotics for several months, depending on the response to treatment [4]. It is reported that doxycycline, rifampicin, and trimethoprim/sulfamethoxazole are useful in the treatment of NB because of its good penetration into the CNS [23]. Treatment regimens, including third-generation cephalosporins with good penetration into the CNS, such as Ceftriaxone, are also available for NB [4]. Our patient was treated with Ceftriaxone, minocycline,e, and rifampicin. The outcome also confirmed the treatment effect.

NB may mimic many central and peripheral nervous system syndromes, which may lead to misdiagnosis and delay treatment. It is an unusual presentation of NB that stroke accompanied chronic encephalomyelitis with paraparesis. NGS can provide an effective and rapid detection method in diagnosis. The fight against this disease should be implemented with a multidisciplinary approach.

## Data Availability

Not applicable.

## Abbreviations

NB: Neurobrucellosis
CSF: Cerebrospinal fluid
MRI: Magnetic resonance imaging
NGS: Next-generation sequencing
TIA: Transient ischemic attack
ELISA: Enzyme-linked immunosorbent assay
PCR: Polymerase Chain Reaction
